# Epstein–Barr Virus Sequence Variations Among the Understudied Nasopharyngeal Carcinoma Patients of Diverse Ancestries in Southeast Asia

**DOI:** 10.1002/jmv.70269

**Published:** 2025-03-05

**Authors:** Hwee Sze Tee, Jingtong Liang, Norazlin Abdul Aziz, Xiang Zhou, Hamidah Akmal Hisham, Ke‐En Tan, Yanhong Chen, Zuriani Burhanuddin, Johnny S. H. Kwan, Kwok‐Wai Lo, Faridah Hassan, Sha'ariyah bt Mohd Mokhtar, Alan Soo Beng Khoo, Miao Xu, Yat‐Yuen Lim, Lu Ping Tan

**Affiliations:** ^1^ Molecular Pathology Unit, Cancer Research Centre, Institute for Medical Research National Institutes of Health, Ministry of Health Malaysia Shah Alam Malaysia; ^2^ State Key Laboratory of Oncology in South China, Guangdong Key Laboratory of Nasopharyngeal Carcinoma Diagnosis and Therapy, Guangdong Provincial Clinical Research Center for Cancer Sun Yat‐sen University Cancer Center Guangzhou China; ^3^ Faculty of Medicine Universiti Teknologi MARA Sungai Buloh Malaysia; ^4^ Department of Prenatal Diagnostic Center, Guangzhou Women and Children's Medical Centre Guangzhou Medical University Guangzhou China; ^5^ Hong Kong Genome Institute Hong Kong China; ^6^ Department of Anatomical and Cellular Pathology, Prince of Wales Hospital The Chinese University of Hong Kong Hong Kong China; ^7^ State Key Laboratory of Translational Oncology, Sir YK Pao Centre for Cancer The Chinese University of Hong Kong Hong Kong China; ^8^ Department of Otorhinolaryngology Selayang Hospital, Ministry of Health Kepong Malaysia; ^9^ Department of Otorhinolaryngology Tengku Ampuan Rahimah Hospital, Ministry of Health Kuala Lumpur Malaysia; ^10^ School of Postgraduate Studies International Medical University Kuala Lumpur Malaysia; ^11^ Department of Medical Oncology, Sidney Kimmel Medical College Thomas Jefferson University Philadelphia Pennsylvania USA; ^12^ Institute of Biological Sciences Faculty of Science Universiti Malaya Kuala Lumpur Malaysia; ^13^ School of Medical and Life Sciences Sunway University Subang Jaya Malaysia

**Keywords:** biostatistics and bioinformatics, Epstein–Barr virus, genetic variation, genetics, virus classification

## Abstract

Epstein–Barr virus (EBV) is associated with cancers, including lymphomas and nasopharyngeal carcinoma (NPC). To date, risk variants for NPC were mainly identified from Chinese populations, which dominated the world's total number of cases. Although Southeast Asia (SEA) countries have among the world's top yet intriguingly diverse NPC age‐standardized incidence rates across subpopulations, data on EBV from SEA remains scarce. In this study, we examined 83 NPC patients of different ancestries for the presence of risk haplotypes associated with the Southern Chinese NPC and generated and analyzed 67 EBV sequences (from tissue, patient‐derived xenografts and lymphoblastoid cell lines of 60 NPC patients) together with 838 published EBV genomes. Our study revealed that NPC patients of non‐Chinese ancestry had fewer risk variants and haplotypes that are associated with Southern Chinese NPC and clustered distinctly from lymphomas, Southern Chinese NPC, and non‐cancer controls. The distribution of non‐synonymous variants was similar among NPC patients of Chinese ancestry, irrespective of geographical location. Meanwhile, non‐synonymous variants in genes related to packaging, latency, and structural proteins such as *BPLF1*, *LF3*, and *LMP1* varied across different ancestries. Our findings suggest possibilities of EBV adaptation to host genetics for NPC pathogenesis and warrant further research for the understudied NPC subpopulations.

## 1 Introduction

Epstein–Barr virus (EBV) is a widespread virus classified in the family *Herpesviridae*. It belongs to the subfamily *Gamaherpesvirinae*, genus *Lymphocryptovirus*, and species *Human herpesvirus 4*. Primary infection often occurs asymptomatically in infants or young children. The virus may persist in a latent form inside resting memory B cells of healthy carriers. EBV is known to cause infectious mononucleosis (an acute mild disease common among young adults), benign or malignant form of the post‐transplant lymphoproliferative disorder (PTLD) and smooth muscle tumors (SMTs) among immunodeficient individuals, Hodgkin's lymphoma, Burkitt's lymphoma, NK/T cell lymphoma, nasopharyngeal carcinoma (NPC), pulmonary lymphoepithelioma‐like carcinoma (PELC), and a subset of gastric carcinoma [1, 2]. Increasing evidence suggests that EBV sequences vary across different populations. There could be dominant strains in specific geographical regions [3], possibly due to genetic drift and selection influenced by human genetic factors such as the human leukocyte antigen (HLA) and EBV genetic factor, including those coding epitope for human T cell recognition [3].

NPC is an epithelial cancer originating from the nasopharynx, with a distinct geographical distribution worldwide. Its development involves multiple factors, including EBV infection, genetic predispositions, and environmental influences [4]. While NPC is exceptionally rare in Western countries, Southeast Asia (SEA) countries have the world's top age‐standardized incidence and mortality rates (ASIRs and ASMRs), and Southern China has reported the highest number of NPC cases yearly [5]. Over the years, host genetic factors and EBV variants conferring high risk for NPC were identified in the Southern Chinese populations [6, 7]. EBV variants in the Japanese population, of which NPC is uncommon, were also reported [8]. Despite having the world's top ASIRs and ASMRs, NPC patients from SEA are still understudied and absent in many large‐scale genomic studies for NPC and EBV. This has hindered a comprehensive investigation, effectively rendering NPC a neglected tropical disease. Notably, significant resources are being directed toward EBV vaccine development, primarily based on data generated from B cell lymphoma and infectious mononucleosis cases in Western countries, as well as NPC cases from Southern China. The scarcity of EBV data from SEA populations who are most affected by EBV‐attributable cancers [9] could further exacerbate health disparities.

In this study, we explore the molecular epidemiology of NPC by investigating the EBV genetic factors that are prevalent in the understudied populations from SEA. Frequencies of three NPC risk variants reported from the Southern Chinese populations were examined in 83 NPC patients of different ancestries for determination of their risk haplotypes. A total of 67 EBV sequences were generated from tumor tissue, patient‐derived xenografts, and lymphoblastoid cell lines of 60 NPC patients via EBV‐targeted capture or whole genome sequencing. Phylogenetic and variant analyses were carried out together with 838 publicly available EBV genomes from different regions and diseases.

## 2 Methods

### 2.1 Patient's Tumor Samples

A total of 86 fresh or formalin‐fixed paraffin‐embedded (FFPE) tissue samples were collected from 83 histologically diagnosed NPC patients. Fourteen NPC PDXs and LCLs generated from eight histologically diagnosed NPC patients from our previous studies [10, 11] were also included in this study. Details of sample type/source and demographics are shown in Table S1. Informed consent was obtained, and the conduct of this study was according to the ethics approved by the Medical Research and Ethics Committee, Ministry of Health Malaysia (NMRR‐18‐596‐41153, NMRR‐11‐461‐9672, NMRR‐18‐264‐40228, NMRR‐12‐1203‐14027, and NMRR‐18‐623‐41161).

### 2.2 DNA Extraction and EBV Load Quantification

Genomic DNA was extracted from cells and frozen or FFPE tissue samples using the QIAamp DNA Mini kit or QIAamp DNA FFPE Tissue Kit, respectively (QIAGEN, the United States). A NanoDrop spectrophotometer was used to determine the concentration of the DNA samples. EBV DNA load was measured by real‐time quantitative polymerase chain reaction (qPCR) toward the BALF5 region [7] using the ABI7500FAST system (Thermo Fisher Scientific, the United States).

### 2.3 Multiplex PCR for *BALF2* Haplotypes Associated With Southern Chinese NPC

Three EBV variants in *BALF2* that are associated with the Southern Chinese NPC [7] (NC_007605.1:162215 C > A, 162476 T > C, and 163364 C > T) were genotyped by multiplex PCR using the Sequenom MassARRAY (Agena Bioscience, Canada) following standard protocols. Samples were classified into having the highest risk (C‐C‐T), high risk (C‐C‐C), wild type (C‐T‐C), or low risk (A‐T‐C) haplotypes.

### 2.4 EBV Genome Sequencing, Assembly, and EBV Typing

A total of 67 EBV sequences were generated from 60 NPC patients by EBV‐targeted captured sequencing or whole genome sequencing of 53 tissue samples from 52 NPC patients and 14 patient‐derived xenografts and lymphoblastoid cell lines from 8 NPC patients. The accuracy of EBV sequencing data was validated by comparison of EBV sequences generated from different technical replicates, including sample replicates for patients with IDs IMR22, B110, G514, G517, and G244 (see Table S1 for details). Detailed steps for sequencing and assembly are listed in Supplementary Methods. Briefly, DNA library prepared from each NPC patient's tumor tissue sample was subjected to paired‐end 150 bp sequencing using NextSeq 500 System (Illumina, the United States). EBV genomes were assembled using Velvet v1.235 and SPAdes v3.15.3 with the EBV genome NC007605.1 as a reference. Assembled genome sizes and details are listed in Table S1.

EBV typing was carried out based on the alignment of *EBNA‐2* and *EBNA‐3* (A, B, and C) sequences from the 67 assembled EBV genomes to the reference Type 1 and Type 2 EBV genomes (Table S2).

### 2.5 Phylogenetic and Variant Analysis

Three approaches were carried out: (a) whole‐genome single nucleotide polymorphism (SNP) analysis; (b) core SNPs; and (c) core‐gene alignment. Variant calling was conducted using Snippy v4.6.0 with default parameters and NC007605.1 as the reference genome. Variant profiles for all 905 EBV genomes (838 publicly available data and 67 NPC EBV genomes generated from this study) were grouped based on the phylogenetic clusters and visualized using R v4.1.0 (Table S3). Detailed steps are listed in Supplementary Methods.

## 3 Results

### 3.1 NPC Risk Haplotypes Identified From the Southern Chinese Populations Are Uncommon in NPC Patients of Non‐Chinese Ancestries

**Figure 1:**
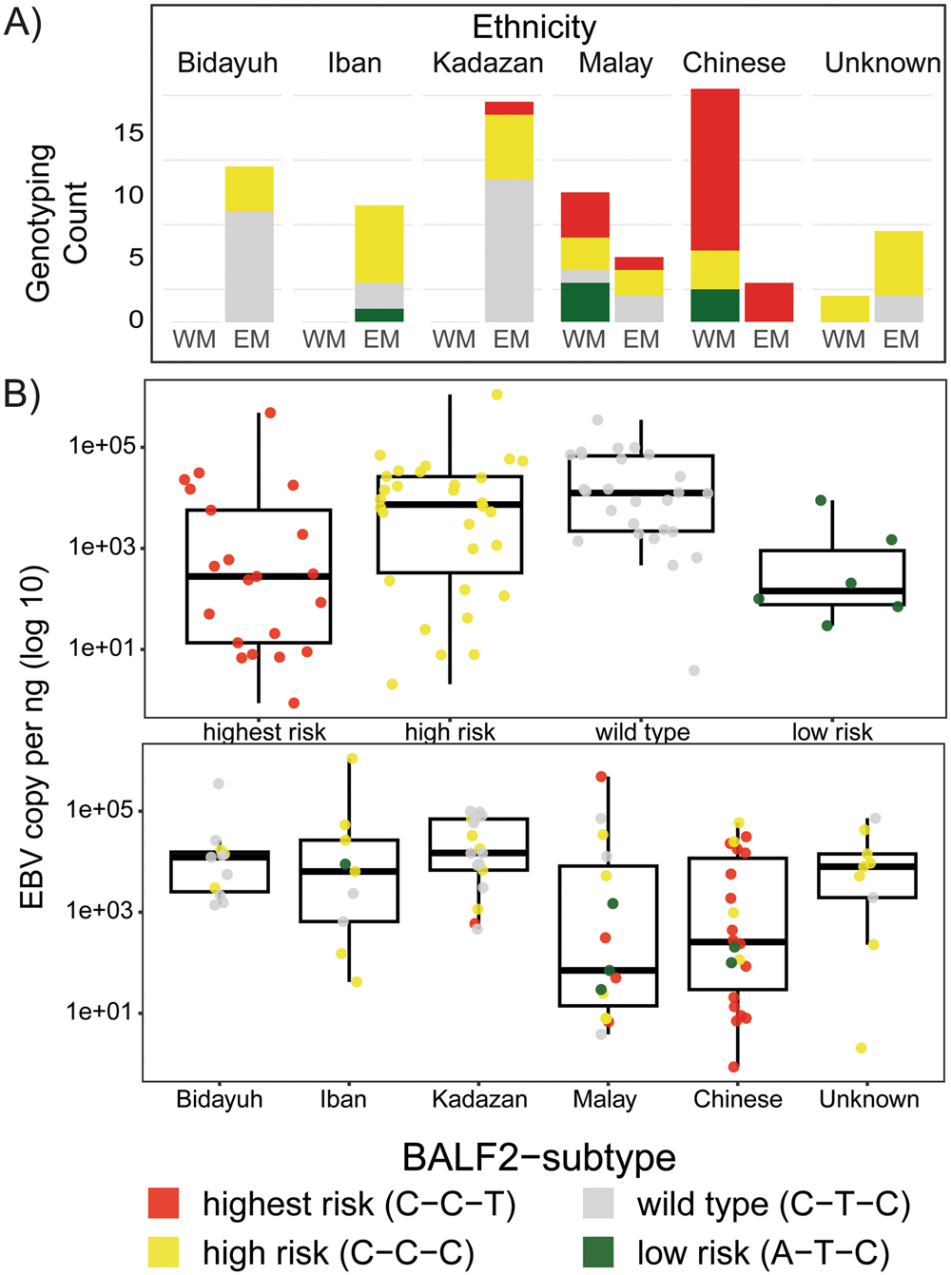
(A) Frequency of *BALF2* haplotypes in 83 NPC patients by ethnicity across East Malaysia (EM) and West Malaysia (WM). (B) Quantitative PCR showing EBV copy per ng of tumor DNA by haplotype (upper panel) and ethnic groups (lower panel).

Malaysia is a multiethnic country with ASIR of NPC ranging from < 1 to > 30 per 100 000 [12]. Different at‐risk groups are unevenly distributed between East Malaysia (EM) and West Malaysia (WM), which are separated by the South China Sea. The natives with the highest ASIRs for NPC (Bidayuh, Kadazan, and Iban) mostly reside in EM, while the Chinese and Malay ethnic groups are found in both regions. Multiplex PCR on 83 NPC patients from these regions revealed that the *BALF2* highest‐risk haplotype for Southern Chinese NPC (C‐C‐T) appeared common only in NPC patients of Chinese ancestry (Figure 1A). Among the 62/83 non‐Chinese NPC patients, 50% (31.5/62) had neither the *BALF2* highest risk (C‐C‐T) nor the *BALF2* high risk (C‐C‐C) haplotypes. Notably, median EBV copy per ng tumor DNA was highest in those with the *BALF2* wild‐type risk (C‐T‐C) haplotype and in native NPC (Figure 1B).

### 3.2 EBVs From NPC Patients of Different Ancestries Clustered Distinctly

**Figure 2:**
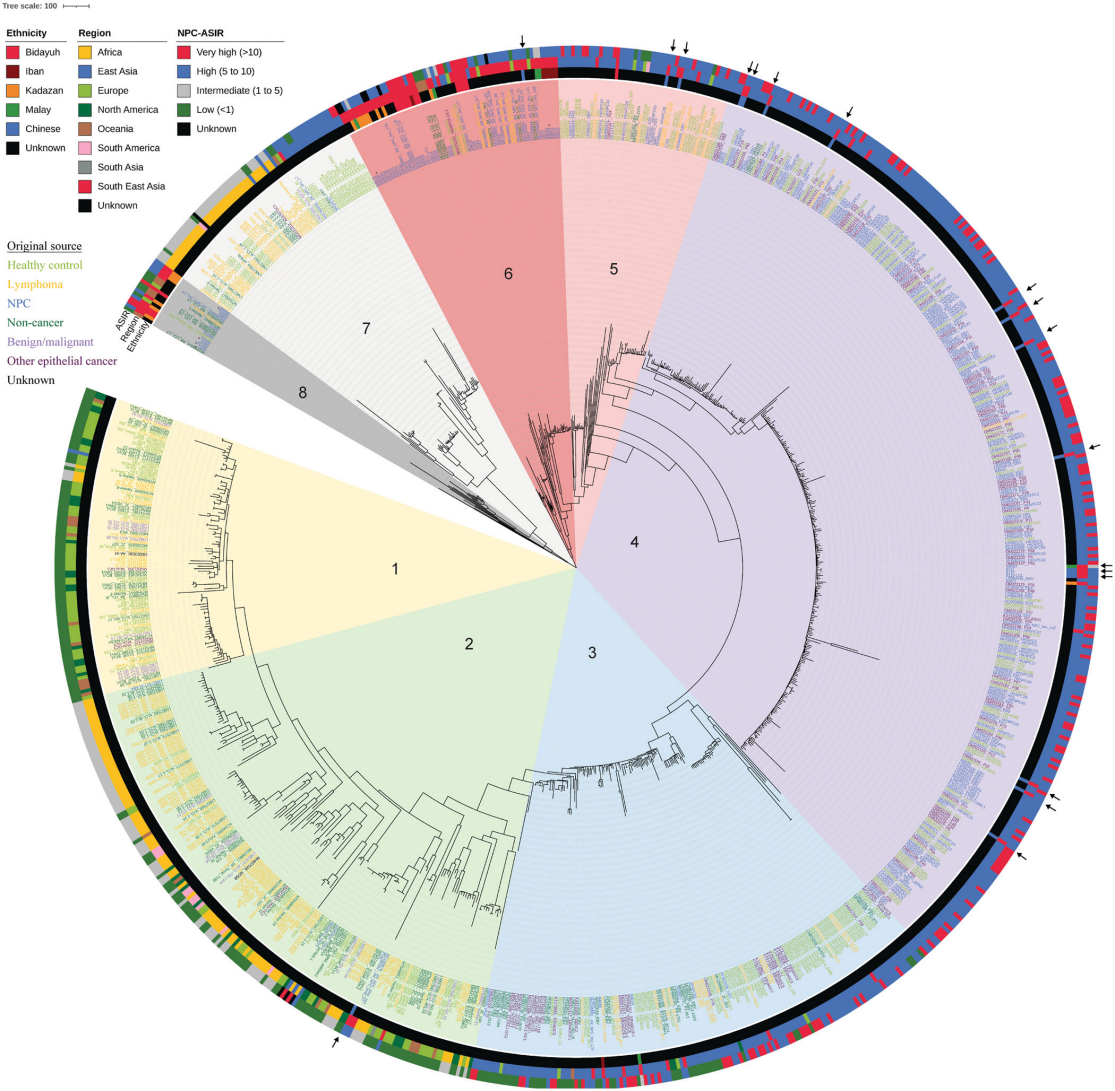
Phylogenetic analysis of 67 EBVs from this study together with 838 publicly available EBV genomes. The latter EBVs were from populations with diverse NPC ASIRs residing in different geographical regions and having different EBV‐associated conditions or diseases. Arrows indicate EBVs derived from Malaysian NPC patients of Chinese ancestry. Tip label color represents the original source of the EBV. The inner circle represents the ethnicity (data only available for samples from this study), the middle circle represents the residing region, and the outer circle represents the NPC ASIR known for the subpopulation (a distinct group within a larger population, typically defined by specific characteristics such as genetic traits, geographical origin, or sociodemographic factors) or country. Labels with an asterisk indicate EBV strains with recombinant type.

Based on the alignment of *EBNA‐2* and *EBNA‐3A*, *3B*, *3C* sequences with the reference Type 1 and Type 2 EBV genomes, it was found that our NPC cohort (total of 67 EBVs from 60 NPC cases) comprises 83% (50/60) Type 1 EBV (in Clusters 2–6), 6.7% (4/60) Type 2 EBV (in Cluster 8), and 10% (6/60) intertypic recombinants (in Clusters 6 and 8) (Figure 2 and Figures S1–S8). Together with whole genome phylogenetic reconstruction of 838 publicly available EBV genomes, we observed that while almost all EBVs from Malaysian NPC patients of Chinese ancestry clustered with EBVs from Southern Chinese NPC patients (Figure 2, Cluster 4), EBVs from Malaysia native NPC patients cluster with others mostly from SEA (Figure 2, Clusters 6 and 8). This result is further supported by core SNPs (Figure S9) and core‐genes alignment (Figure S10) phylogenetic analyses.

### 3.3 Known NPC Risk Associated EBV Variants and Non‐Synonymous Variants in Selected EBV Genes Had Varying Distribution Patterns Across NPC Patients of Different Ancestry Backgrounds

**Figure 3:**
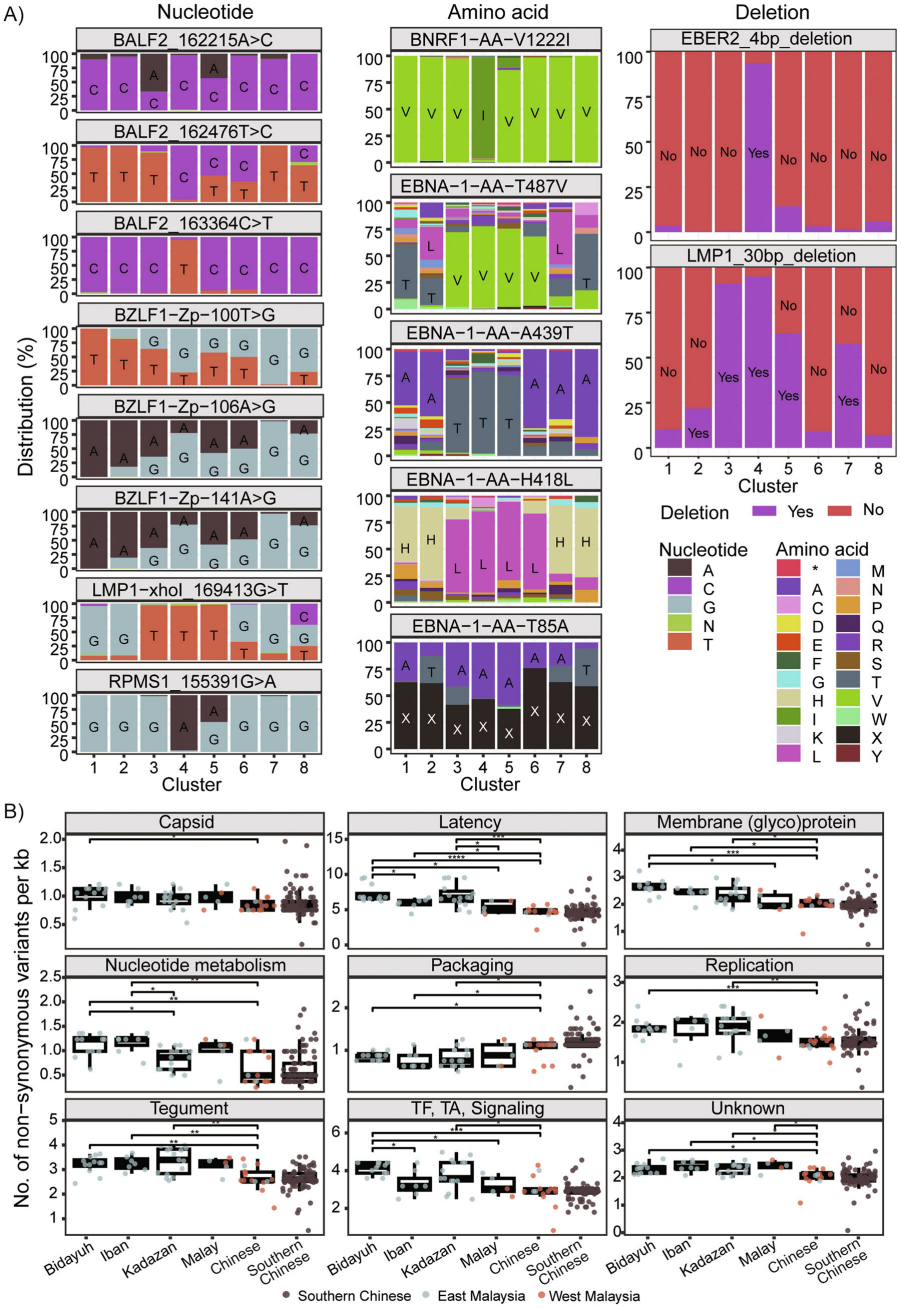
Distribution of known risk variants and non‐synonymous variants among EBV genomes. (A) Distribution of previously reported NPC risk variants among 905 EBV genomes. Color represents different nucleotides/amino acids or the presence/absence of deletions in the genes among the EBV clusters. (B) Number of non‐synonymous variants per kilobase gene length among EBV genes grouped by functions. EBV genomes from 60 NPC patients of various ancestries were compared to those from the 95 Southern Chinese NPC patients. Dot color represents patients' location. Asterisks indicate statistical significance (Benjamini–Hochberg‐adjusted *p* value) between groups. * *p* ≤ 0.05; ** *p* ≤ 0.01; *** *p* ≤ 0.001; **** *p* ≤ 0.0001. Abbreviations: TF, transcription factor; TA, transactivator.

Analysis revealed that aside from *BALF2* variants, some of the known NPC‐risk variants discovered from the Southern Chinese populations, such as *EBER2*‐deletion (*EBER*‐del) [6], *RPMS1*_G155391A [13], and *BNRF1*‐V1222I [14], and variants reported to be higher in NPC, including *BZLF1* Zp‐V3 variant (G‐G‐G) [15], *EBNA‐1* T85A, A439T, and H418L [16], and *LMP1* XhoI G169413T and 30‐bp deletions [17], are indeed common in Cluster 4 but less or uncommon in Clusters 6 and 8 (Figure 3A).

Analysis of all non‐synonymous variants based on EBV genome NC007605.1 (constructed from EBVs originating from B cells) showed that non‐synonymous variants are detected in many genes, sometimes the percentages varied drastically across different clusters (Figure S11). Among the NPC‐enriched clusters, deleterious variants of *BPLF1, LMP1*, and *RPMS1* were more frequently found in Cluster 4 as compared to Clusters 6 and 8, while the opposite pattern was observed with *LF3* (Figure S12).

When only Southern Chinese NPC and Malaysian NPC samples with ethnicity information were analyzed and the EBV genes grouped by functions, it was observed that, generally, the group of EBV genes with latency function had the highest numbers of non‐synonymous variants per kilobase as compared to EBV genes of other functions (Figure 3B). This finding suggests that latency‐related functional differences could be the most common differences between EBV derived from NPC and EBV derived from B cells (reference genome used in this study). Interestingly, NPC patients of Chinese ancestry had similar EBV variant distribution patterns irrespective of residing regions (Figure 3B). Meanwhile, compared to NPC patients of Chinese ancestry, NPC patients of non‐Chinese ancestry harbored a significantly lower number of non‐synonymous variants per kilobase in the packaging‐related group of EBV genes and the highest number of non‐synonymous variants per kilobase in the latency, tegument, membrane (glyco)protein, nucleotide metabolism, transcription factors, transactivators, and signaling‐related groups of EBV genes (Figure 3B).

## 4 Discussion

In our cohort of 83 NPC patients of different ancestries, we examined the presence of *BALF2* haplotypes that are associated with the Southern Chinese NPC. We carried out a phylogenetic and in‐depth variant analysis of 67 EBV sequences generated from our study together with 838 publicly available EBV genomes from individuals with different EBV‐associated conditions or diseases, residing in different regions. Our findings revealed that EBVs of NPC patients of different ancestries form distinct clusters with unique non‐synonymous variants distribution patterns in selected EBV genes and may have different EBV functional properties.

Similar to the *BALF2* haplotypes that are associated with the Southern Chinese NPC, we found that *BPLF1*, *LMP1*, *EBER2*, *BALF2*, *RPMS1*, *BZLF1*, and *BNRF1* variants and deletions that are more frequently reported in Southern Chinese NPC patients are uncommon in our cohort of non‐Chinese NPC patients. This is in line with the reports that *RPMS1*_G155391A and *EBER* variants that are common among Southern Chinese NPC patients were uncommon in Indonesian NPC patients [18], while the *BALF2* highest risk haplotype (C‐C‐T) for Southern Chinese NPC was absent in Japanese NPC patients [8]. Perhaps another hint of EBV adaptation to the host's genetic background is the variations that we observed among the native NPC patients residing within the same region. The *BZLF1* Zp‐V3 variants are prevalent among Indonesians, Ibans, and Malays but not among Bidayuhs and Kadazans. *EBNA‐1*‐A439T that is common among Indonesians are uncommon among Malaysian natives. Among the Malaysian native NPC, the *BALF2* highest risk haplotype (C‐C‐T) for Southern Chinese NPC (red in Figure 1) is absent in the Bidayuhs and Ibans but present in 5.9% (1/17) of Kadazan NPC patients (Table S1). This may be explained by anecdotal evidence of relatively more common and increasing intermarriages between the Kadazan and Chinese, known as sino‐kadazans in Malaysia [19, 20]. Nonetheless, the unavailability of human genomic data to infer genetic admixture and the small sample size in the current study limits further interpretation. Future studies on genetic and historical factors may provide clearer insights into these observations.

Recently, a global analysis of EBV phylogeny and gene variants that included nearly 200 novel EBV genomes from different disease phenotypes of previously understudied populations in Central and South America and East Africa cautioned that the previously identified disease‐specific variants could be limited and biased by the available genomes from specific regions [21]. This, together with findings from our study, elicits questions about the inference of previously reported high‐risk EBV variants for Southern Chinese NPC patients [6, 7, 13, 14, 17] in the context of global NPC. Further investigations in large cohort studies, especially from the understudied populations with the world's top NPC ASIRs, such as those from Brunei, Maldives, Indonesia, Malaysia, and Vietnam [5], are required.

The clustering of EBVs based on similarity in the host's ancestry irrespective of the host's residing geographical region suggests specific host–pathogen interactions and selective pressure on EBV infection by the host. To our knowledge, our study is the first to compare the possible impact on functional properties by all non‐synonymous variants in EBV across NPC patients of different ancestries. The non‐synonymous EBV variants that showed different distribution patterns across ancestries in this study (Figure S11 and Figure 3B) could affect viral packaging, latency, transcription factors, transactivators, and signaling, as well as potentially cause changes in epitopes that may lead to immune evasion [14]. Consistent with a previous study that showed EBV genes related to latency are undergoing stronger positive selection [18], we also find these genes being the most affected in NPC not only upon comparison to EBV from B cells but also across NPC patients of different ancestries (Figure 3B). Given the observed lower tumor EBV DNA load in NPC patients of Chinese ancestry (Figure 1B) and the lower salivary EBV DNA load that has been reported in Southern Chinese NPC patients, it is interesting to speculate that the higher viral packaging gene variants observed in them could be affecting viral load, which reflects a delicate equilibrium between host immunity and viral adaptation to evade immune surveillance. On the other hand, the significantly higher percentage of variants in EBV genes related to latency, membrane glycoproteins, nucleotide metabolism, tegument, transcription factors, transactivators, and signaling among the Bidayuh, Kadazan, and Iban populations suggests that the virus has evolved distinct mechanisms to adapt to these specific host populations and may confer advantages in terms of persistence and transmission. To examine this hypothesis, large case–control studies of these understudied populations and more comprehensive functional studies on selected high‐risk variants using EBV‐positive NPC models of different genetic backgrounds are required to elucidate and validate the mechanisms underlying EBV and human host–cell interactions.

In summary, our findings show that EBVs from NPC patients of different ancestries could be genetically distinct. The marked difference in the relative prevalence of EBV variants between people of different ancestries, even from within the same country, highlights the importance of molecular epidemiological studies of EBV and ancestry‐matched controls. The addition of 67 EBV sequences from our understudied population to the publicly available set of EBV genome sequences will enable future meta‐analysis of NPC‐specific EBV variants and provide new insights into EBV adaptation and/or co‐segregation with human host cells and immune evasion. In conclusion, our study highlights the possible need for population‐specific approaches to improve accuracy in risk prediction and EBV targeting strategies for the prevention and treatment of EBV‐related diseases.

## Author Contributions

**Hwee Sze Tee:** EBV genome assembly, data analysis, interpretation, co‐writing the manuscript. **Norazlin Abdul Aziz:** histopathology, sample preparation. **Jingtong Liang:** EBV sequencing, genotyping, quality control of data. **Yanhong Chen:** EBV sequencing, genotyping, quality control of data. **Xiang Zhou:** EBV sequencing, genotyping, quality control of data. **Hamidah Akmal Hisam:** salivary EBV load qPCR, assist in EBV variant analysis. **Ke En Tan:** salivary EBV load qPCR, assist in EBV variant analysis. **Zuriani Burhanuddin:** salivary EBV load qPCR, assist in EBV variant analysis. **Johnny SH Kwan:** generation of whole genome sequences. **Kwok‐Wai Lo:** generation of whole genome sequences. **Faridah Hassan:** samples and clinical data collection. **Sha'ariyah bt Mohd Mokhtar:** samples and clinical data collection. **Miao Xu:** conception of the study design, EBV sequencing and genotyping analysis. **Alan Khoo Soo Beng:** data interpretation and critical review of the manuscript. **Yat Yuen Lim:** data analysis, interpretation, co‐writing the manuscript. **Lu Ping Tan:** conception of the study design, sample preparation, data analysis, interpretation, co‐writing the manuscript.

## Conflicts of Interest

The authors declare no conflicts of interest.

## Supporting information

Supporting information.

Supporting information.

## Data Availability

The data that support the findings of this study are available on request from the corresponding author. The data are not publicly available due to privacy or ethical restrictions.
