# Supplementary material for: Epstein–Barr Virus Sequence Variations Among the Understudied Nasopharyngeal Carcinoma Patients of Diverse Ancestries in Southeast Asia
Source: J Med Virol. 2025 Mar 5;97(3):e70269. doi: 10.1002/jmv.70269 (PMC11881214; doi:10.1002/jmv.70269)
Supplement: Supplementary file 2 — Supporting information. [file JMV-97-e70269-s001.docx]

Supplementary Materials for

**Epstein-Barr virus sequence variations among the understudied Nasopharyngeal Carcinoma patients of diverse ancestries in Southeast Asia**

**Hwee Sze Tee, Jingtong Liang, Norazlin Abdul Aziz, Xiang Zhou, Hamidah Akmal Hisham, Ke-En Tan, Yanhong Chen, Zuriani Burhanuddin, Johnny SH Kwan, Kwok-Wai Lo, Faridah Hassan, Sha'ariyah bt Mohd Mokhtar, Alan Soo Beng Khoo, Miao Xu, Yat-Yuen Lim, Lu Ping Tan**

The PDF file includes:

Supplementary Methods

Supplementary Figures 1-12

References

Other Materials for this manuscript include the following:

Supplementary Tables 1-4

**Supplementary Methods**

**Generation of EBV sequences from NPC samples**

A total of 67 EBV sequences were generated from 60 NPC patients. Among these, 53 were tumour tissue samples from 52 NPC patients and 14 were in-house PDXs and LCLs from 8 NPC patients. Data was generated via in-house (n = 53) or outsource (n = 10) EBV-targeted capture sequencing or derived from whole genome sequencing (n = 4). Details are listed in Supplementary Table 1.

For in-house EBV-targeted capture sequencing, 200 ng of DNA was fragmented, purified, end blunted, adaptor ligated, and amplified by the NadPrep EZ DNA Library Preparation Module (for Illumina®). The DNA library was quantified using Qubit (Thermo Life Qubit 3.0) and verified by Qsep1. The final constructed DNA fragments were around 300–500 bp. Pretreated DNA was then subjected to hybrid capture using the EBV-targeting single-stranded DNA probes (Integrated DNA Technologies). After capture enrichment with NadPrep® Hybrid Capture Reagents, libraries were sequenced (paired-end 150 bp) using the NextSeq 500 System (Illumina, San Diego, USA).

For outsource EBV-targeted capture sequencing, 500 ng of DNA was sent for EBV targeted capture sequencing with the MyGenostics EBV genome capture system.

For whole genome sequencing, DNA was examined with agarose gel electrophoresis and quantified using Quant-IT PicoGreen (Invitrogen, USA). The sequencing libraries were prepared with TruSeq Nano DNA Library Prep Kit (Illumina, #FC-121-4003) according to the manufacturer’s instructions. Briefly, 100ng of genomic DNA was fragmented using adaptive focused acoustic technology (AFA; Covaris). The fragmented DNA was repaired with ‘A’ ligated to the 3′ end followed by ligation of Truseq adapters to the fragments and then PCR amplification. The final purified product was quantified with qPCR and qualify assessed by the 4200 TapeStation system. (Agilent technologies, USA). Paired-end sequencing was carried out using the HiSeqX platform (Illumina, San Diego, USA).

**Genome assembly of EBV sequences**

Genome assemblies of the four NPC PDXs with whole genome sequencing data (B110, B111, G514, G517) were created using Velvet v1.235^1^ and VelvetOptimiser v2.2.5^1^, scaffolded using SSPACE v2.1.1^2^, contigs ordered using Ragout v2.0^3^ with the EBV B95.8+Raji genome^4^ (NC007605.1) as reference, and gaps filled using GapFiller v2.1.1^5^ and GapCloser v1.12^6^.

EBV-targeted capture sequencing data from 63 samples in this study and publicly available EBV genome sequencing data of 4 Japanese NPC samples were examined for sequence quality and filtered using fastp v0.23.2^7^. Filtered reads were mapped to the EBV NC007605.1 genome using BWA v0.7.17^8^ and duplicate reads were marked using PICARD tool v2.25.1^9^. Reads were then assembled using Velvet v1.235^1^, VelvetOptimiser v2.2.5^1^, and SPAdes v3.15.3^10^ with default and meta modes, and the best assemblies were selected based on QUAST v5.0.2^11^ output. Contigs were further scaffolded using SSPACE v2.1.1^2^, aligned, and ordered using ABACAS v1.3.1^12^.

**Phylogenetic analysis**

To study the phylogenetic relationship among the EBV genomes, three approaches were carried out: a) whole genome single-nucleotide-polymorphism (SNP) analysis; b) core SNPs; and c) core-gene alignment. For whole genome SNPs, 1,370 EBV genomes were downloaded from the NCBI Virus Genome Database (accessed on May 2023). Genomes with sizes <100kb were removed and were subjected to deduplication using BBMap v38.90^13^ with identity=99%. The final set of 905 genomes (Supplementary Table 3) were subjected to variant calling with default parameters and gene annotations were carried out with NC_007605.1 genome^4^ used as reference. Repetitive regions were masked to generate whole genome alignment using Snippy v4.6.0 (<https://github.com/tseemann/snippy>).

For core SNPs and core-genes alignment (n=97), a smaller set of reference genomes (n=26) were used together with EBV genomes assembled in this study (n= 67 generated from this study + 4 from the published Japanese study = 71). Core SNP alignment was generated using snippy-core from Snippy v4.6.0. For core-genes alignment, genes were first annotated using Geneious Prime v2022.1.1 (https://www.geneious.com) with B95-8+Raji (NC007605.1) and AG876 (DQ279927.1) as references. Core genes that were used for species tree reconstruction were identified using OrthoFinder v2.5.5^14^ with parameter blastp identity=70% and inflation=1.2. After manual filtration and verification, 79 single-copy genes were individually aligned using MAFFT v7.475^15^ and concatenated to generate a core-genes alignment. Phylogenetic trees of whole genome SNPs, core SNPs, and core-genes alignment were then reconstructed using the ultrafast bootstrap approximation in IQTree v2.1.3^16^.

**EBV Variant analysis**

Variants were shortlisted into non-synonymous-variants (by excluding intron, synonymous, non-coding transcript, or intergenic region variants) and deleterious-variants (retaining only frameshift, stop-gained or disruptive-in-frame variants). Results were visualized using R v4.1.0^17^. Variant frequency was calculated for each cluster with the following formula:

Number of samples with variants x 100 %

Total number of samples

The functions of EBV genes were categorized according to literature^18^ and listed in Supplementary Table 4. The burden of non-synonymous variants on each functional group for each unique NPC case was calculated with the following formula:

Total number of non-synonymous variants in all genes in functional group X

Total gene length in Kilobase for all genes in functional group X

**Supplementary Figures**


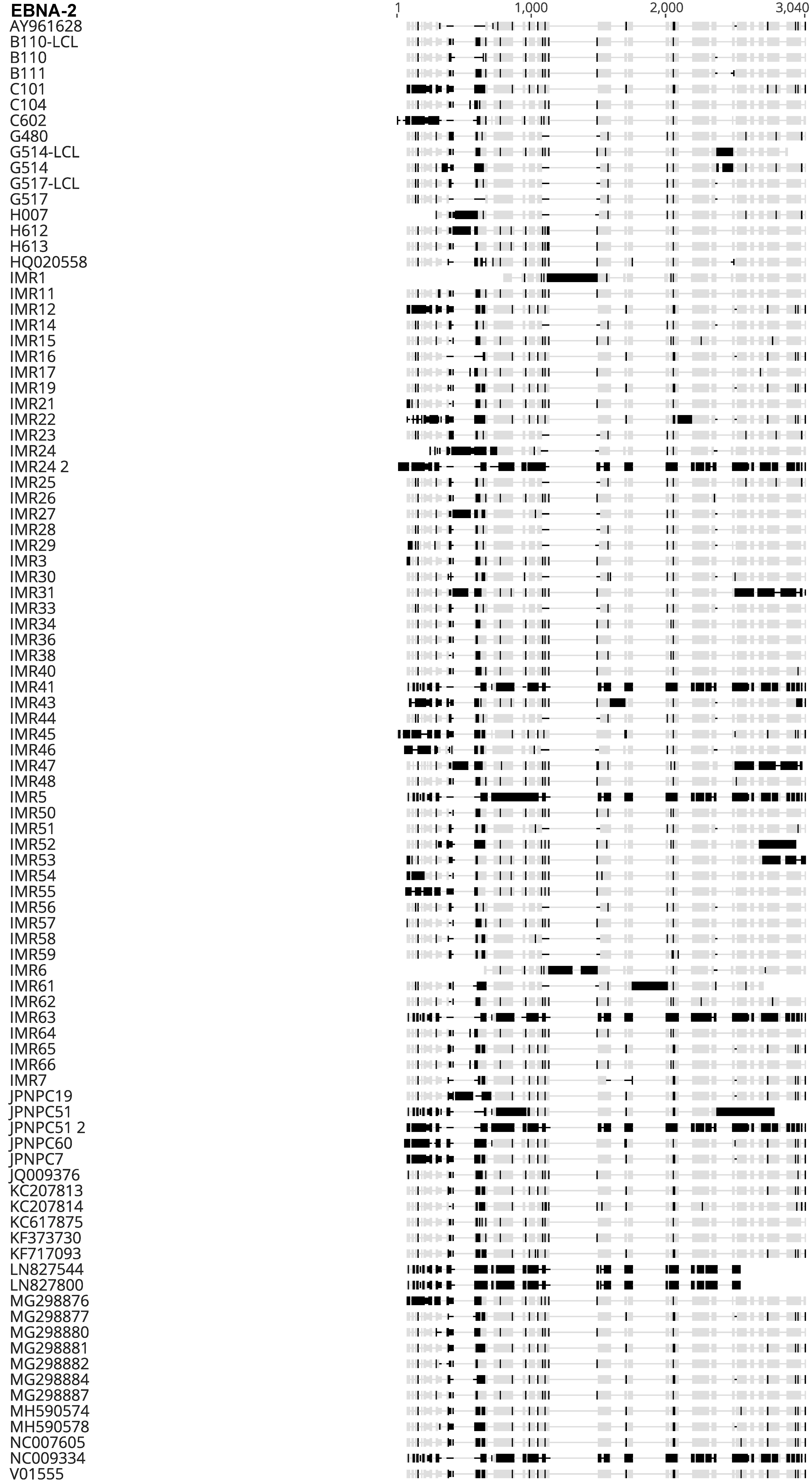


Supplementary Figure 1. MAFFT-alignment of *EBNA-2* genes from studied EBV genomes.


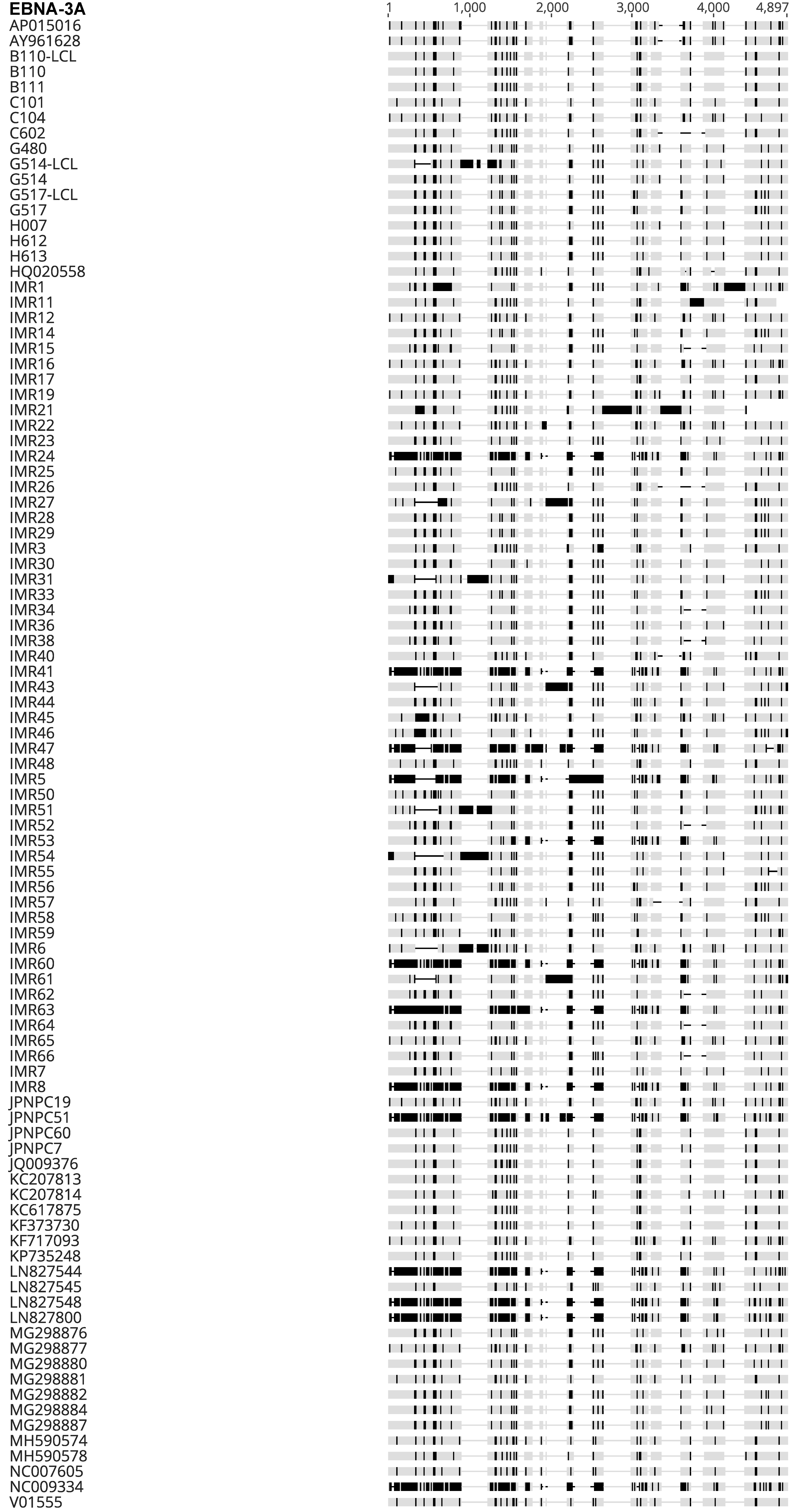


Supplementary Figure 2. MAFFT-alignment of *EBNA-3A* genes from studied EBV genomes.


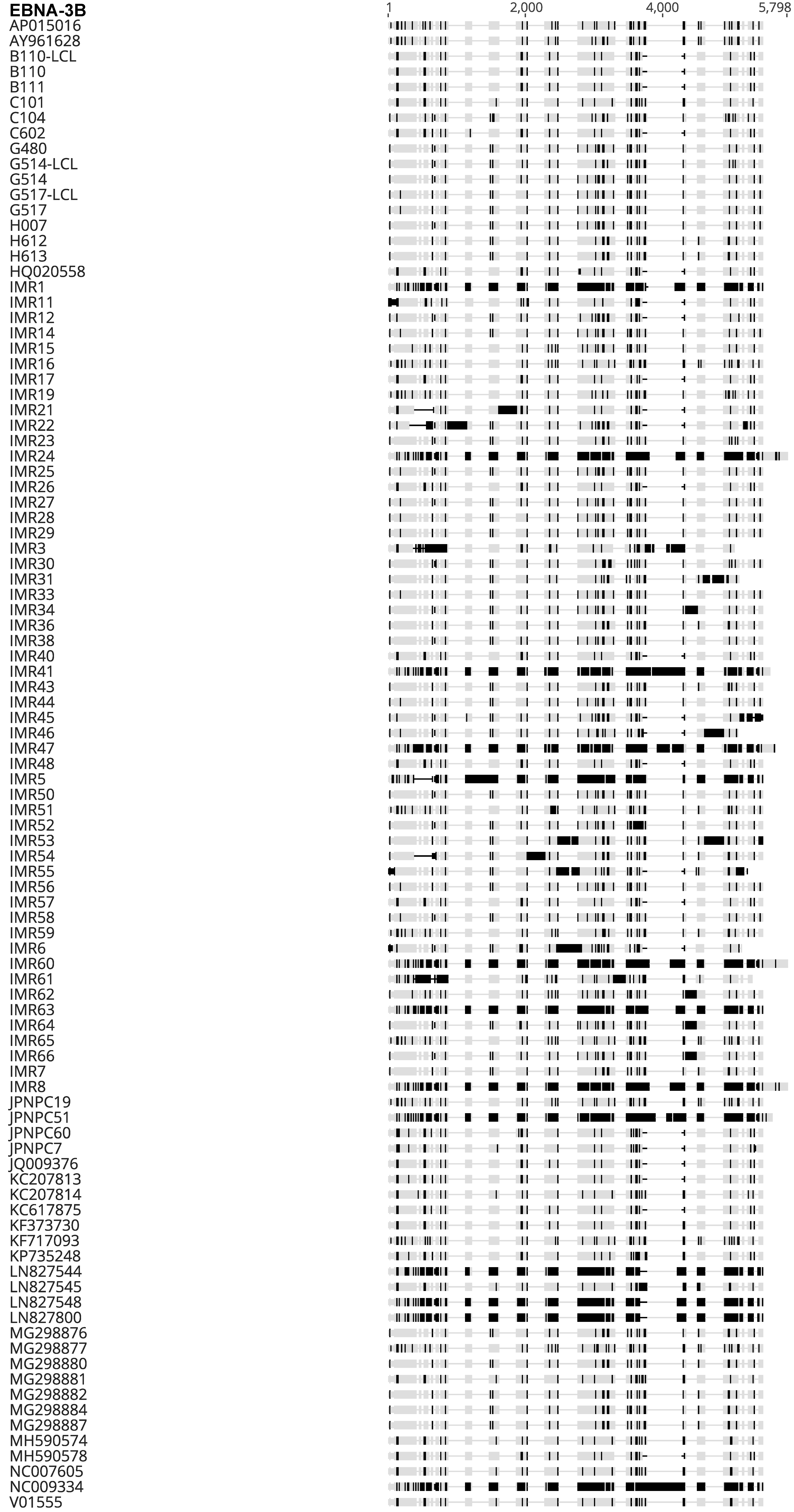


Supplementary Figure 3. MAFFT-alignment of EBNA-3B genes from studied EBV genomes.


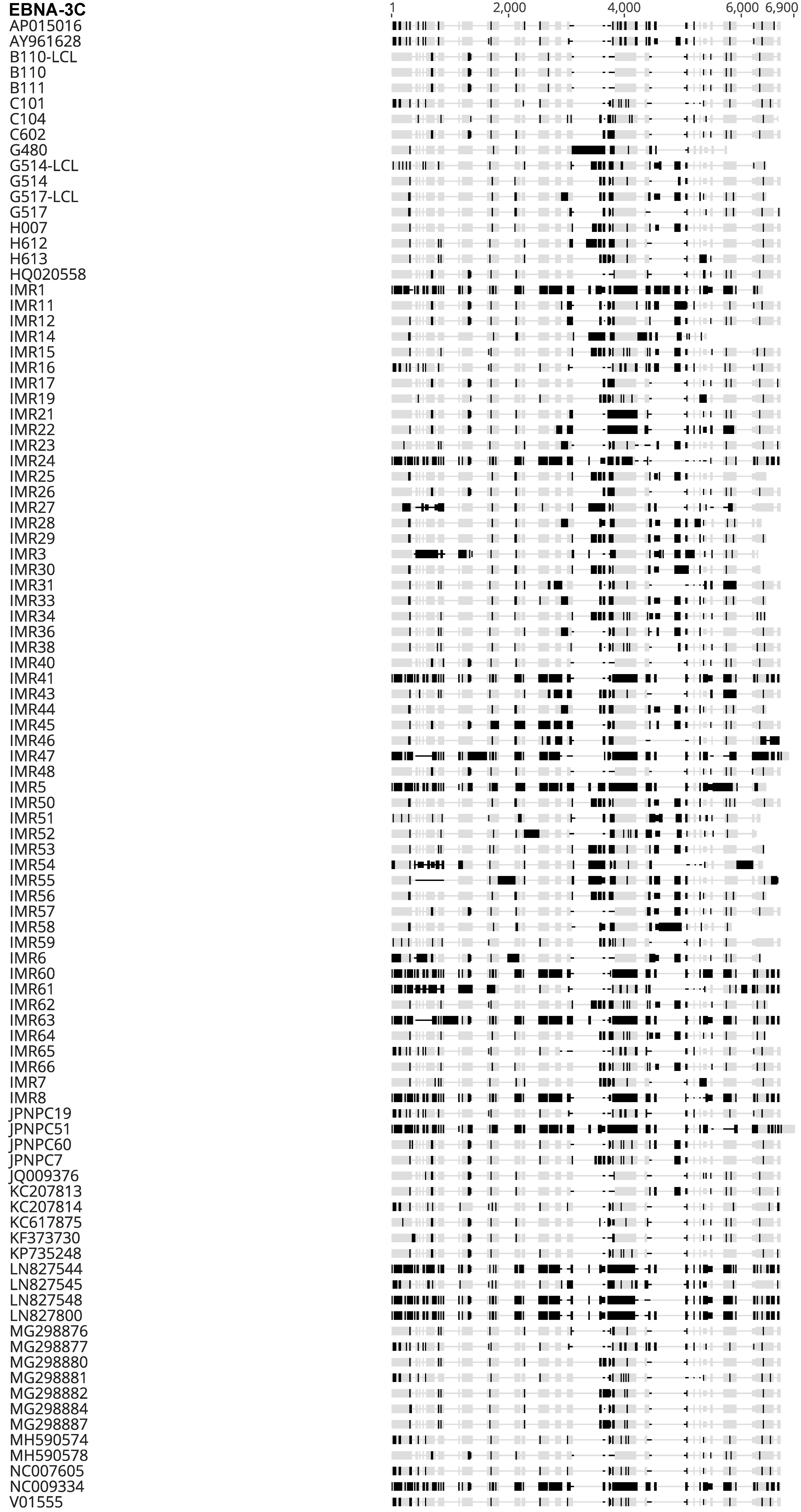


Supplementary Figure 4. MAFFT-alignment of *EBNA-3C* genes from studied EBV genomes.


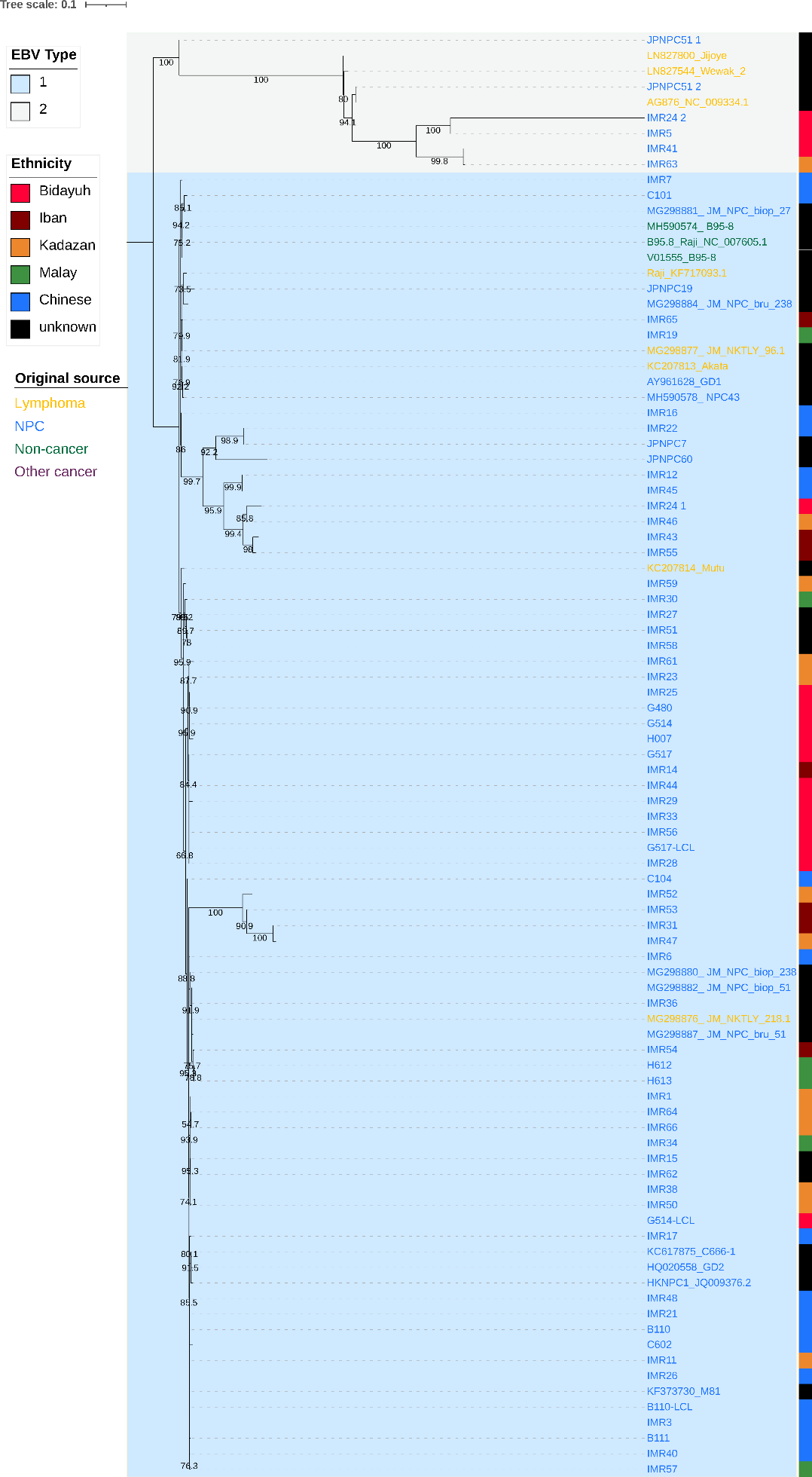


Supplementary Figure 5. Phylogenetic tree of *EBNA-2* genes from studied EBV genomes reconstructed using IQTree. Background color represents EBV types and tip label color represents the original source of EBV. The rightmost column with color indicates the ethnicity of the samples.


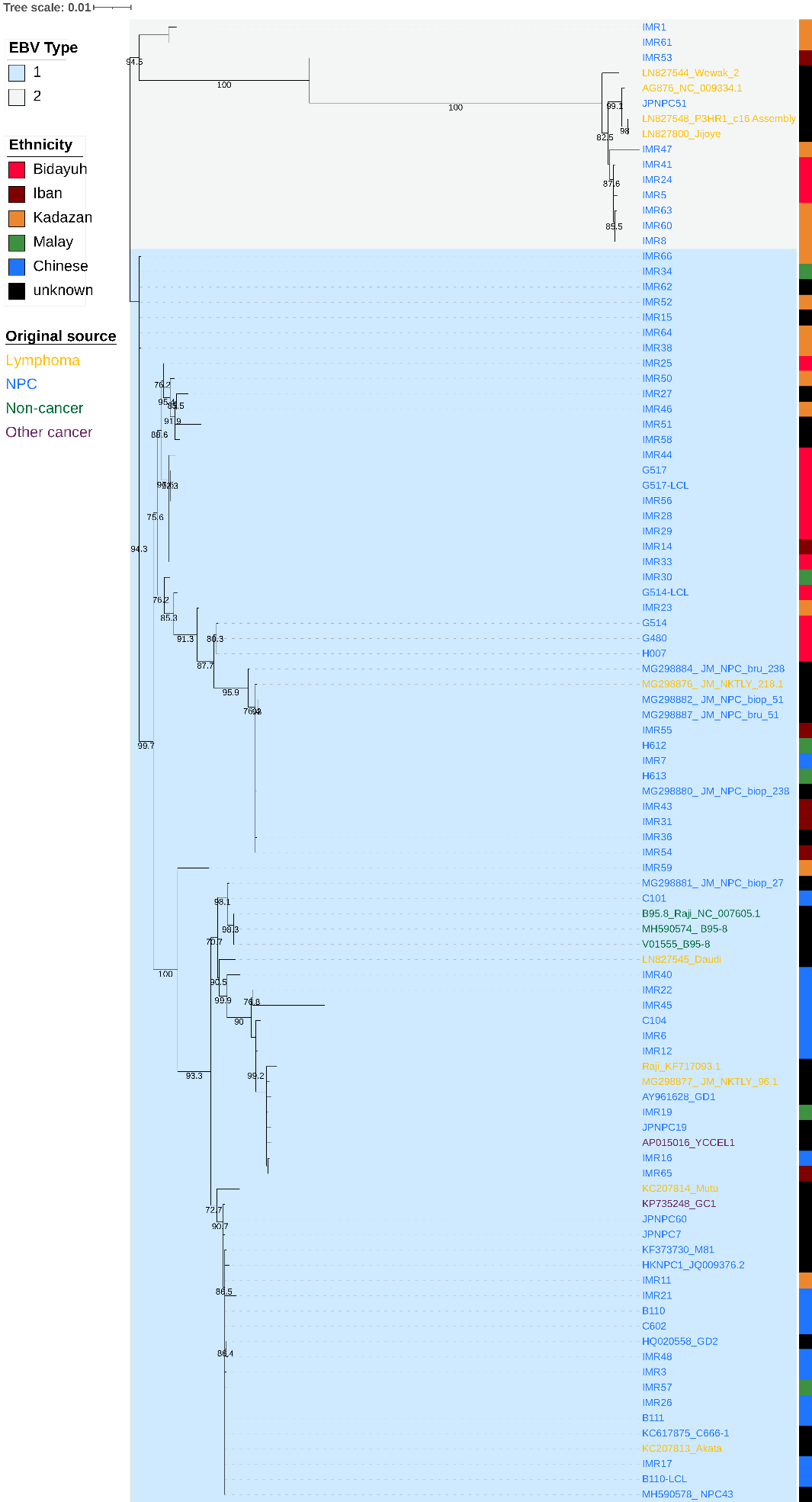


Supplementary Figure 6. Phylogenetic tree of *EBNA-3A* genes from studied EBV genomes reconstructed using IQTree. Background color represents EBV types and tip label color represents the original source of EBV. The rightmost column with color indicates the ethnicity of the samples.


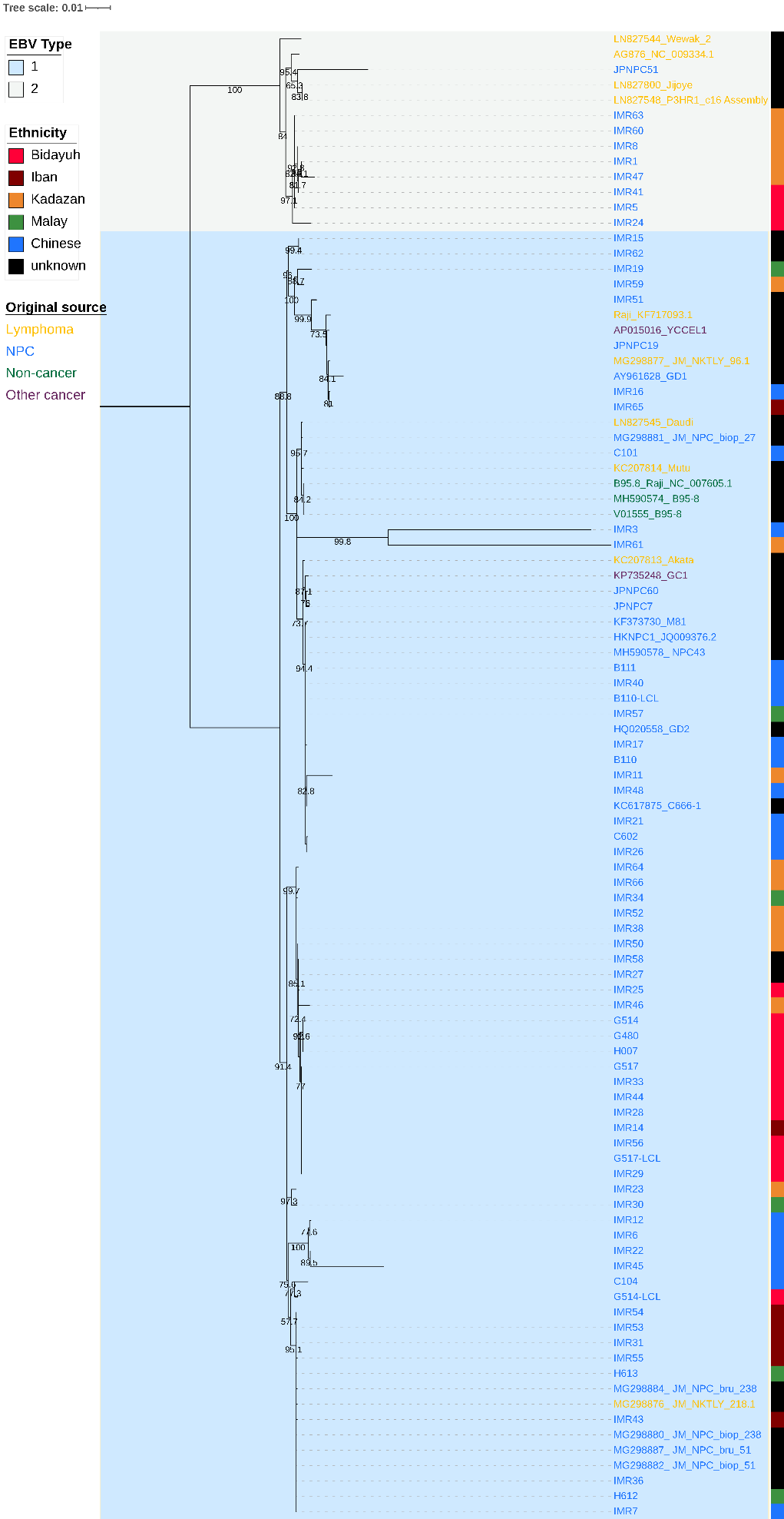


Supplementary Figure 7. Phylogenetic tree of *EBNA-3B* genes from studied EBV genomes reconstructed using IQTree. Background color represents EBV types and tip label color represents the original source of EBV. The rightmost column with color indicates the ethnicity of the samples.


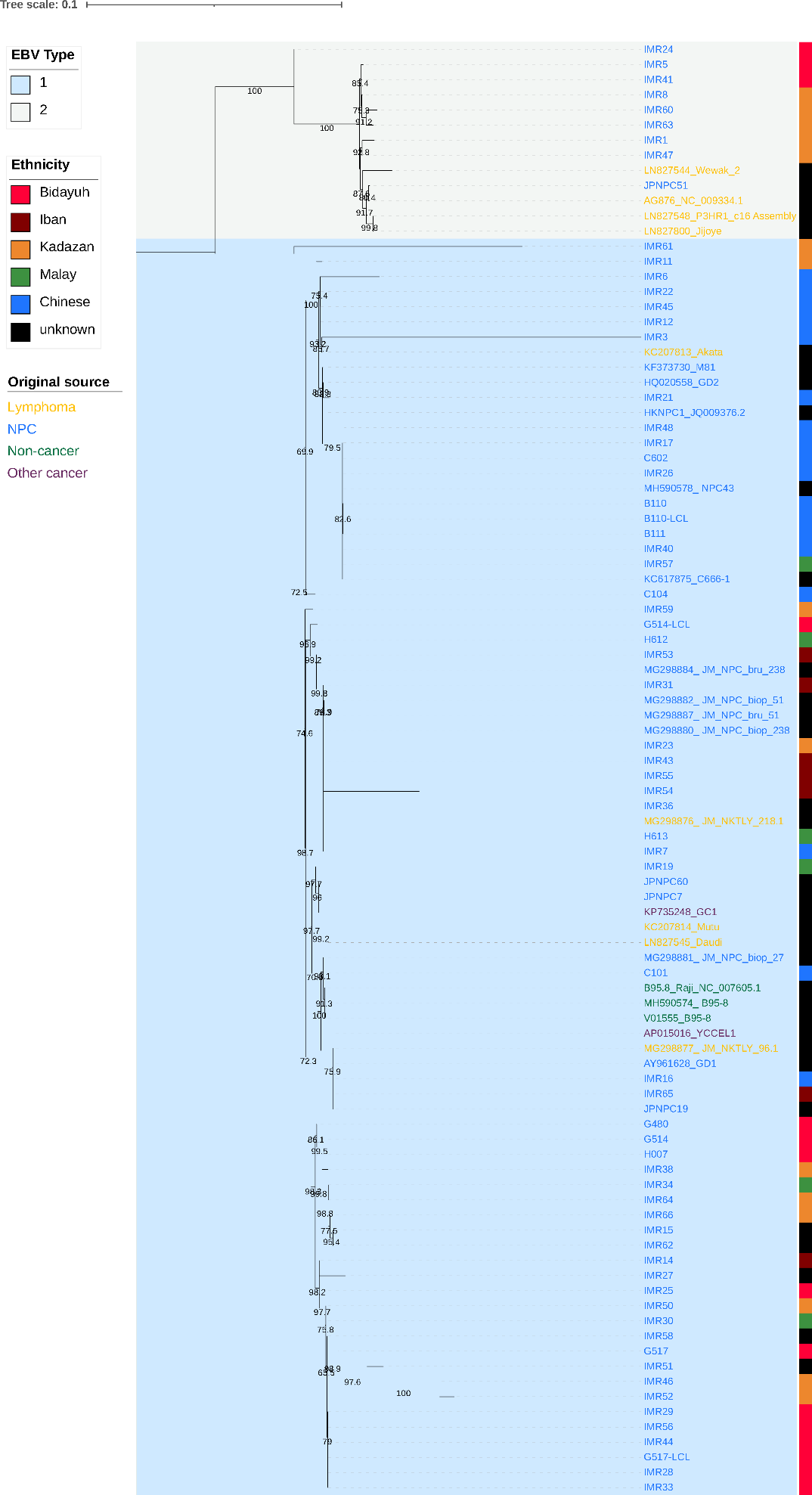


Supplementary Figure 8. Phylogenetic tree of *EBNA-3C* genes from studied EBV genomes reconstructed using IQTree. Background color represents EBV types and tip label color represents the original source of EBV. The rightmost column with color indicates the ethnicity of the samples.


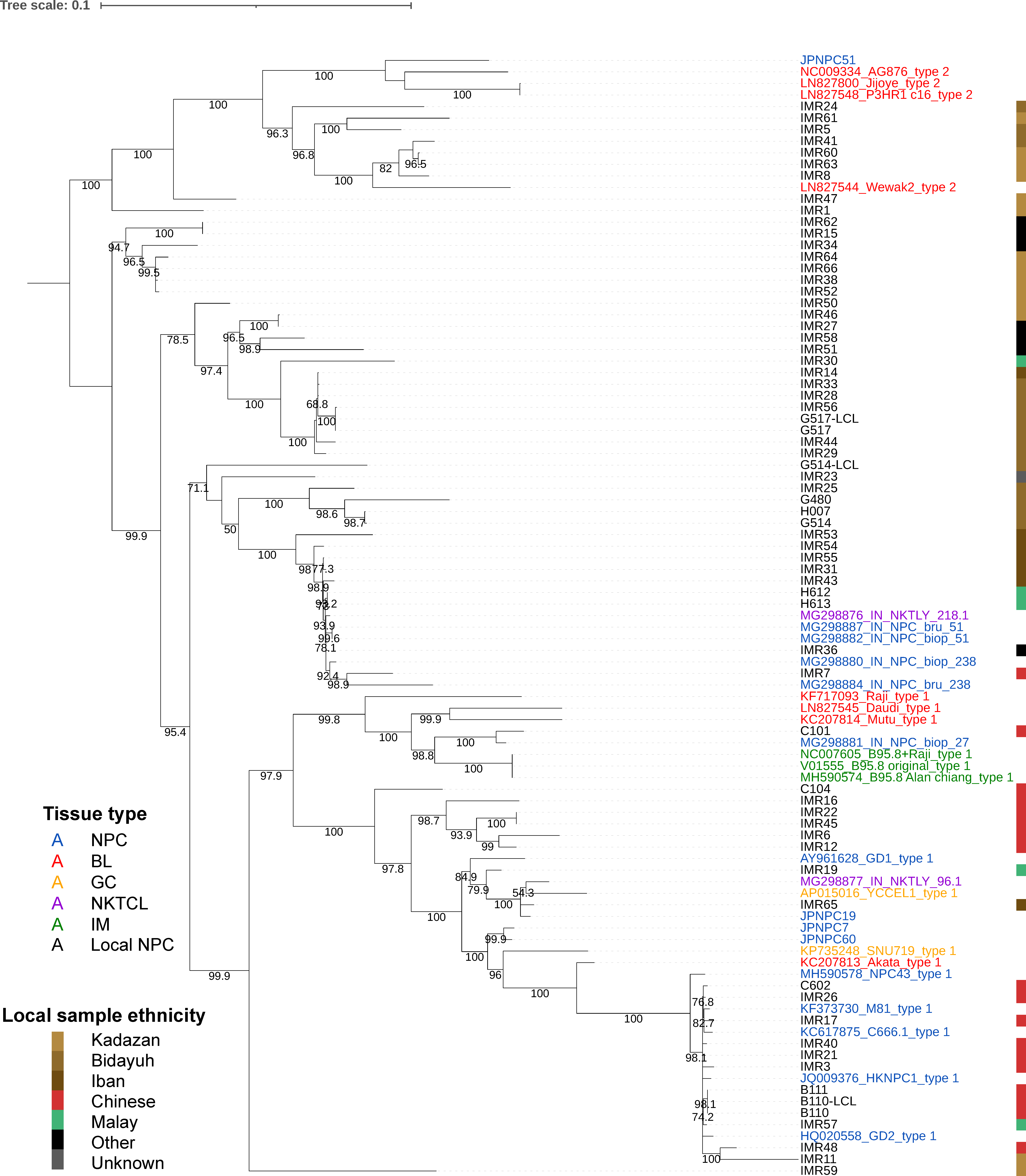


Supplementary Figure 9. Phylogenetic tree based on 2,460-bp core SNPs alignment of 97 EBV genomes using NC_007605.1 B95.8+Raji EBV as reference. The tree is rooted to Type 2 EBV genomes based on the reference EBV strains. Tip label color represents the original source where the EBV genome was derived from and filled-square color indicates the ethnicity of local NPC patient. Scale bar represent the number of substitutions per site and bootstrap values over 50% are shown.





Supplementary Figure 10. Phylogenetic tree based on 79 core-genes alignment shared among 97 EBV genomes using NC_007605.1 B95.8+Raji EBV as reference. The tree is rooted to Type 2 EBV genomes based on the reference EBV strains. Tip label color represents the original source where the EBV genome was derived from and filled-square color indicates the ethnicity of local NPC patient. Scale bar represent the number of substitutions per site and bootstrap values over 50% are shown.





Supplementary Figure 11. Frequency of EBV with (green) or without (red) non-synonymous variants by cluster.





Supplementary Figure 12. Frequency of EBV with (green) or without (red) deleterious variants by cluster.

**References**

1. Zerbino DR, Birney E. Velvet: Algorithms for de novo short read assembly using de Bruijn graphs. *Genome Res*. 2008;18(5):821-829. doi:10.1101/gr.074492.107

2. Boetzer M, Henkel CV, Jansen HJ, Butler D, Pirovano W. Scaffolding pre-assembled contigs using SSPACE. *Bioinformatics*. 2011;27(4):578-579. doi:10.1093/bioinformatics/btq683

3. Kolmogorov M, Raney B, Paten B, Pham S. Ragout-a reference-assisted assembly tool for bacterial genomes. *Bioinforma Oxf Engl*. 2014;30(12):i302-309. doi:10.1093/bioinformatics/btu280

4. Miller G, Shope T, Lisco H, Stitt D, Lipman M. Epstein-Barr virus: transformation, cytopathic changes, and viral antigens in squirrel monkey and marmoset leukocytes. *Proc Natl Acad Sci U S A*. 1972;69(2):383-387. doi:10.1073/pnas.69.2.383

5. Boetzer M, Pirovano W. Toward almost closed genomes with GapFiller. *Genome Biol*. 2012;13(6):R56. doi:10.1186/gb-2012-13-6-r56

6. Luo R, Liu B, Xie Y, et al. SOAPdenovo2: an empirically improved memory-efficient short-read de novo assembler. *GigaScience*. 2012;1(1):2047-217X-1-18. doi:10.1186/2047-217X-1-18

7. Chen S, Zhou Y, Chen Y, Gu J. fastp: an ultra-fast all-in-one FASTQ preprocessor. *Bioinformatics*. 2018;34(17):i884-i890. doi:10.1093/bioinformatics/bty560

8. Li H, Durbin R. Fast and accurate short read alignment with Burrows–Wheeler transform. *Bioinformatics*. 2009;25(14):1754-1760. doi:10.1093/bioinformatics/btp324

9. Broad Institute. Picard Toolkit. Published online 2019. https://broadinstitute.github.io/picard/

10. Prjibelski A, Antipov D, Meleshko D, Lapidus A, Korobeynikov A. Using SPAdes De Novo Assembler. *Curr Protoc Bioinforma*. 2020;70(1):e102. doi:10.1002/cpbi.102

11. Gurevich A, Saveliev V, Vyahhi N, Tesler G. QUAST: quality assessment tool for genome assemblies. *Bioinforma Oxf Engl*. 2013;29(8):1072-1075. doi:10.1093/bioinformatics/btt086

12. Assefa S, Keane TM, Otto TD, Newbold C, Berriman M. ABACAS: algorithm-based automatic contiguation of assembled sequences. *Bioinformatics*. 2009;25(15):1968-1969. doi:10.1093/bioinformatics/btp347

13. Bushnell B. BBMap: A fast, accurate, splice-aware aligner. Published online March 19, 2014. https://sourceforge.net/projects/bbmap/

14. Emms DM, Kelly S. OrthoFinder: phylogenetic orthology inference for comparative genomics. *Genome Biol*. 2019;20(1):238. doi:10.1186/s13059-019-1832-y

15. Katoh K, Standley DM. MAFFT multiple sequence alignment software version 7: Improvements in performance and usability. *Mol Biol Evol*. 2013;30(4):772-780. doi:10.1093/molbev/mst010

16. Minh BQ, Schmidt HA, Chernomor O, et al. IQ-TREE 2: New models and efficient methods for phylogenetic inference in the genomic era. *Mol Biol Evol*. 2020;37(5):1530-1534. doi:10.1093/molbev/msaa015

17. R Core Team. R: a language and environment for statistical computing. Published online 2023. https://www.R-project.org

18. Tarbouriech N, Buisson M, Géoui T, Daenke S, Cusack S, Burmeister WP. Structural genomics of the Epstein–Barr virus. *Acta Crystallogr D Biol Crystallogr*. 2006;62(10):1276-1285. doi:10.1107/S0907444906030034
